# Hong Kong young people’s blood donation behavior

**DOI:** 10.4103/0973-6247.76000

**Published:** 2011-01

**Authors:** Juliana Hong, Alice Yuen Loke

**Affiliations:** *Doctoral Candidate, Hong Kong Polytechnic University*; 1 *Professor, Hong Kong Polytechnic University, Hong Kong*

**Keywords:** Blood donation behavior, socio-demographic factors, Voluntary blood donor; Donor retention

## Abstract

**Introduction:**

Similar to other developed countries, only 3% of the total population in Hong Kong donate blood (Hong Kong Red Cross Blood Transfusion Service 2003). More than 20% of annual donations come from youngsters aged 18-25. However, this category of donors has decreased gradually from 24.6% in 2004 to 22.9% in 2008. This study aims to examine the characteristics and intention of young blood donors versus nondonors in Hong Kong; and to explore the factors that may influence Hong Kong young people’s donation behavior.

**Materials and Methods:**

This is a cross-sectional study using questionnaire to solicit information from young people including both blood donors and non-donors.

**Results:**

It showed that more non-donors were underweight (26%) than blood donors (16.9%). Blood donors demonstrated to have more knowledge on the usage of donated blood (87.2%). Nearly half of youngster admitted that they made use of donation as a means for blood testing (53.1%) or free physical check-up (47.3%).

**Conclusions:**

Recruitment strategies should focus on the enhancement of health education programs related to blood and blood donation for young people to increase their awareness of blood and alleviate their misconceptions about blood donation.

## Background

Blood is a vital healthcare resource; thus, a reliable and sufficient blood supply is of paramount importance in healthcare setting and blood donors are always the primary focus of blood transfusion service. Similar to many countries over the world, only 3% of the total population in Hong Kong donate blood.[1] More than 20% of annual donations come from the category of young adults aged 18 to 25. However, percentage of the total donations from this group of donors has decreased gradually from 24.6% in 2004 to 22.9% in 2008.

According to the Hong Kong Government projection, 27% of the population will be over 65 years of age by 2033.[2] The effects of an aging population will become a crisis as early as 2013, when many baby boomers reach the age of 60. On the other hand, Hong Kong’s birth rate is one of the lowest compared to the other cities in the world. Due to the steep decline in fertility, the number of students donating blood in schools dropped by 7.3% from 43,844 in the 2006/2007 school year to 40,647 in the 2007/2008 school year.[3] It is estimated that the availability of blood donors in Hong Kong is getting very limited in the next decades.

Besides, a total of 451,781 patients aged 70 and above were admitted into the hospitals under Hospital Authority in 2008 and the increase was 7.1% over the preceding year.[4] Simultaneously, 75,809 units of red blood cell were transfused to the patients aged 70 or over, showing an increase of 3% when comparing with 2007.[5]

### Study aims

The objectives of this paper are (1) to examine the characteristics and intention of young blood donors versus non-donors in Hong Kong; (2) to explore the factors that influencing Hong Kong young people’s donation behaviors.

## Materials and Methods

### Design

As information about Hong Kong young people’s attitudes and behavior toward blood donation is limited, a cross-sectional survey was performed to profile the current situation. A structured self-administered questionnaire was used to solicit information from young people.

### Instrument

A newly refined and structured questionnaire was developed. The “theory of planned behavior”[6] was adopted as the theoretical basis with the inclusion of variables examining Hong Kong young people’s behaviors toward blood donation.

### Respondents

Young Chinese people aged 18 to 25, including both blood donors and non-donors, were the targeted population of this study. The donors were recruited from multiple blood donation centers, and the non-donors were recruited from schools/ institutes including those who accompanied their friends to the centers but did not donate blood [Table 1].

**Table 1:** Demographic characteristics of young adult participants (*n*=3316)

Demographics	Blood donors (*n*=2523)		Non-donors (*n*=785)		P-value*
	*n*	%	*n*	%	
Age					<0.0001
18-20	1218	48.4	452	59.7	
21-25	1300	51.6	305	40.3	
Mean age (SD)	20.91	(2.25)	20.36	(2.15)	<0.0001
Gender					0.372
Males	979	39.1	309	41.0	
Females	1523	60.9	445	59.0	
Body size (BMI)					<0.0001
Underweight (≤ 18.4)	428	18.1	199	28.9	
Normal weight (18.5-22.9)	1408	59.6	378	54.9	
Over weight (23.0-27.5)	434	18.4	95	13.8	
Obese (≥ 27.6)	91	3.9	17	2.5	
Birthplace					<0.0001
Hong Kong	2123	84.9	584	77.7	
Mainland China	319	12.8	135	18.0	
Macau and others	59	2.4	33	4.3	
Years resided in HK					<0.0001
Less than five years	34	1.3	36	4.6	
Six-ten years	129	5.1	58	7.4	
Over 10 years	2274	90.1	676	86.1	

* *P*-value of Fisher exact test for binary variable or independent *t*-test for continuous variable

## Results

### Socio-demographic profile

A total of 3,316 young people were recruited in August and September 2008. The response rate was 94.7%. Among the respondents, 2,523 (76.1%) were blood donors (BD) and 785 (23.7%) were non-donors.

Blood donors in this study were statistically significant heavier (57.9 kg) than nondonors (55.3 kg) with *P*<0.0001. They showed to have a higher BMI (81.9% normal weight or above) than nondonors (71.1% with *P*<0.0001). Non-donors were significantly underweight (28.9%) and 22.3% of them were born outside Hong Kong. They also resided in Hong Kong for only a short period of less than 10 years (12.0%) [Table 2].

**Table 2:** Previous blood donation behaviors and experience among blood donors (n=2523)

	n	%
Last blood donation		
Within one year	6	0.3
Two to five years ago	2217	94.7
Six years or more	119	5.0
Number of times giving blood		
Once	597	24.0
Two–five times	1344	54.1
Six–ten times	362	14.6
Over 10 times or more	182	7.3
Most common venue attending for blood donation		
Donor center	1819	73.4
Mobile donation team	633	25.5
Blood vehicle	27	1.1
Accompanied by friends when donating blood	1471	59.2
Ever experienced adverse reaction from blood donation		
None	1859	73.7
Dizziness	246	9.8
Faint without injury	37	1.5
Faint with injury	13	0.5
Bruise / hematoma	225	8.9
Nausea and vomiting	43	1.7
Feeling fatigue after donation	83	3.3
Others	6	0.2
Satisfied with venepuncture technique of staff at blood donation venue	2236	91.0
Satisfied with the attitude of staff at blood donation venue	2329	94.8
The environment of blood donation venue is pleasant and comfortable	2230	90.8

^*^P-value of Fisher exact test for binary variable or independent *t*-test for continuous variable

### Previous blood donation behavior and experience

Among the blood donors, more than half of them had two to five donations (54.1%); however, only 3% of them donated blood within an interval of one year. Blood donors used to donate blood at static donor centers (73.4%). Majority of blood donors did not encounter any adverse reaction during donation (73.7%). About one tenth of blood donors experienced mild reactions including dizziness (9.8%), bruise/hematoma (8.9%), or feeling fatigue (3.3%) in their blood donations. A small proportion of donors came across severe reaction that had led to subsequent injuries such as fatally faint (0.5%). Majority of blood donors satisfied with the BTS’ services (staff techniques 91%, staff attitudes 94.8%, environment of donation venues 90.8%). However, some of them disagreed with the long waiting time for donating blood (18.9%).

### Knowledge and perception about blood donation

Blood donors demonstrated better knowledge on blood and blood donation related issues than the non-donors. Most of blood donors knew their blood type (ND: 91.8% *vs* ND: 16.1%, *P*<0.0001), the usage of donated blood (BD: 87.2% *vs* ND: 82.9%) and realized that blood was needed to save life (BD: 94.8% *vs* ND: 90.9%). Blood donors perceived they had possessed better health (BD: 96.6% *vs* ND: 90.6%) and less likely practised in high-risk behaviors (BD: 95.7% *vs* ND: 92.7%). Blood donors more actively participated in other kind of donation than non-donors. Many of them had registered as organ donors (BD: 33.8% *vs* ND: 17.2%, *P*<0.0001). Unfortunately, a small proportion of respondents revealed that they had donated blood even though they engaged in high-risk behavior such as having sex with strangers (1.3%) and more than one sexual partner (1.0%).

Nearly one third of non-donors indicated that they would make use of blood donation as a means for a free physical check-up (29.5%) and blood testing of HIV / Hepatitis B (22.9%). They also agreed more with the barriers impacting their blood donation behavior such as afraid of pain (45.8%), afraid of needle (34.3%), afraid of sight of blood (20.3%), too thin (22.5%), afraid of being fainted when donating blood (15.5%), having no time (21.6%), afraid of affecting their health (14%).

### Perception toward BTS

A statistically significant high percentage of blood donors agreed with blood transfusion service’s (BTS) good reputation (91.8%), professional knowledge (95.6%) and excellent service (94.2%) than those of non-donors (78.3, 76.8 and 71.1% respectively, all with *P*<0.0001). However, some blood donors agreed with the poor service provided by BTS which included the long waiting time for blood donation (18.9%, *P*=0.036) and the inconvenient operation hours of donation centers (15.4%, *P*=0.012). Non-donors shared similar findings as they also considered it, the long waiting time for donation (15.5%) and the inconvenience of BTS’ operating hours (19.4%). The results also revealed a clear negative effect that respondents disagree with being informed by BTS of not suitable for blood donation (BD: 9.4% and ND: 15% with *P*<0.0001).

### Attitudes

Blood donors had more positive attitude toward blood donation than non-donors. Blood donors agreed that donating blood was very rewarding as people will reciprocate to them whenever they were in need (BD: 81.6% *vs* ND: 74.1%) and blood donation was beneficial to their health (BD: 61.2% *vs* ND: 51.9%). However, respondents also indicated that they had not been requested by other people for blood donation (BD: 53.4% *vs* ND: 38.1%). Blood donors considered their blood type was important (BD: 37.9% *vs* ND: 25.6%). They also agreed with their friends’ expectation for blood donation (BD: 32.8%) than non-donors (24.3%, *P*<0.0001).

### Social norms

Blood donors considered blood donation was a kind of social responsibility (83.3%) and moral obligation (64.9%). Besides, more blood donors had family members donating blood (BD: 29.4% *vs* ND:19.3%). In contrast, non-donors disagreed that they had a duty to donate blood (72.7%) and blood donation was the moral obligation (48.1%, *P*=0.0001).

### Perceived behavioral control

Blood donors show significantly stronger control power than non-donors. Non-donors concern more about the barriers than blood donors which include “they are not sure about the donation procedures” (BD: 7.3% *vs* ND: 35%), “afraid of needles” (BD: 18% *vs* ND: 34.3%), “being too thin” (BD: 6.7% *vs* ND: 22.5%), “having no time” (BD: 11.1% *vs* ND: 21.6%), “being afraid of the sight of blood” (BD: 9.7% *vs* ND: 20.3%), “would faint if donate blood” (BD: 4.9% *vs* ND: 15.5%) and “donation may affect their health” (BD: 3.9% *vs* ND: 14%) [Table 3].

**Table 3:** Hong Kong young people’s knowledge and perception of blood donation

Demographics	Blood donors (*n*=2523)		Non-donors (*n*=785)		*P*-value*
	*n*	%	*n*	%	
Knowledge about own blood type					<0.0001
O+	1007	40.3	47	6.4	
A+	512	20.5	30	4.1	
B+	603	24.1	23	3.1	
AB+	131	5.2	12	1.6	
Negative blood groups	40	1.6	6	0.7	
Do not know	205	8.2	616	83.9	
Family members ever received blood transfusion	453	18	140	18.6	0.706
Knowing the usage of donated blood	2197	87.2	624	82.9	0.003
Knowing blood is needed to save life	2390	94.8	687	90.9	<0.0001
Registered as organ donor	845	33.8	129	17.2	<0.0001
Knowledge about appropriate interval for blood donation					
For males: Every three months					<0.0001
Correct	1831	77.3	447	63.4	
Incorrect	537	22.7	258	36.6	
For females: Wait at least four to six months					<0.0001
Correct	1996	83.2	458	65.1	
Incorrect	402	16.8	245	34.9	
Self-perceived health status at present					<0.0001
Very good or good	2307	96.6	651	90.6	
Fair or poor	83	3.4	68	9.5	

* P-value of Fisher exact test for binary variable or independent t-test for continuous variable

### Intention

Blood donors demonstrated significantly greater intention to give blood. The majority of blood donors indicated that they had decided to donate (BD: 83.6% *vs* ND: 42.5%), having the intention to donate (BD: 76.3% *vs* ND: 48.5%) or having intention to try (BD: 74.7% *vs* ND: 61%). More blood donors also agreed with being influenced by BTS information to donate blood (BD: 47.7% *vs* ND: 33.5%) (all with *P*<0.0001).

## Discussion

Most of the blood donors are heavier in weight (59.6%) than the non-donors. Besides, a significant underweight condition among young people has been identified in this study. This will eventually affect the eligibility of young people for blood donation in future. The study results also show that non-donors tend to be much lighter and more than one forth of them are underweight (28.9%) whereas the percentage of underweight non-donors is double than that of donors in 21 to 25 years range for both males and females. Since body weight is one of the crucial criteria for blood donation, youngsters of low body weight will be excluded for donation of blood.

Blood donors prefer to make their donation at static donation centers (73.4%) than at mobile collection teams (25.5%) and blood donation vehicles (1.1%). However, non-donors consider donating blood is inconvenient as it takes a long waiting time (15.5%). They also find the location of donation centers and their operation hours unsuitable (28.8 and 19.4% respectively). Findings from a previous Hong Kong study indicated that there was a problem dealing with those blood donors who preferred the door-to-door service and were only willing to donate blood during the visits of the mobile collection teams to their workplace or near their home.[7]

In Hong Kong, a large number of potential donors were lost due to temporary pre-donation deferral every year. There were 32,014 potential donors deferred in 2009. Among them, 50.6% were rejected because of low hemoglobin level, 11.0% were under medical care and 9.7% were not feeling well at the time before donation. The results of this study also indicate that a certain percentage of young people admit that they have been informed by BTS for not suitable to give blood (BD: 9.4% and ND: 15%). Among these young people, particularly the non-donors, temporary deferral may psychologically lead to an excuse for not donating in future[8] and interventions are therefore required to alleviate these negative impacts.

Although a significant number of young people understand the usefulness of blood donation (BD: 87.2%, ND: 82.9%) and also know that blood is needed for saving life (BD:94.8%, ND: 90.9%), it remains unknown why the non-donors of this category lacks of willingness to donate. A lack of relevant information about blood or blood donation is reported to be a cause of keeping many young people away from blood donation, and it is commonly used by the non-donors as an excuse of not giving blood.

Some non-donors indicate that, even though their family members had received blood transfusion in the past (18.6%), they never ever donate. The above data let us to infer that strategies have to be formulated to encourage this group of individuals to reciprocate those in need.

On the other hand, blood donors point out that they expect people to reciprocate them whenever they are in need (BD 81.6% and ND 74.1%). This finding implies that blood donation behavior may not be driven out of pure altruism – the behavior that only concerned the welfare of others regardless of personal benefits.[9] Blood replacement programs can be introduced to encourage those recipients’ relatives to replenish and donate some blood.

The majority of blood donors in the current study perceive they are able to overcome obstacles and barriers for blood donation without great difficulty. In consistent with the previous researches,[10–14] non-donors more agree with the barriers. Other than promotional strategies, elimination of barriers is crucial to improving people’s participation in blood donation. The BTS can try to implement some interventions such as installation of audiovisual equipment in the donation venues, increase of water intake prior to donation[15] to remedy the situation.

## Conclusion

Young people in fact do not oppose the idea of donating blood to save lives. Yet motivating people to donate blood is undoubtedly a difficult task. Provision of effective health education programs from BTS to the general public is one of the crucial factors in recruiting and retaining blood donors. BTS can convey updated knowledge to the young people to increase the awareness of blood donation and alleviate the misconceptions about blood donation among the general public.
